# Multimorbidity, healthy lifestyle, and the risk of cognitive impairment in Chinese older adults: a longitudinal cohort study

**DOI:** 10.1186/s12889-023-17551-1

**Published:** 2024-01-02

**Authors:** Xiaolong Xing, Xueli Yang, Jinqian Chen, Jin Wang, Bowei Zhang, Yanrong Zhao, Shuo Wang

**Affiliations:** 1 Tianjin Key Laboratory of Food Science and Health, School of Medicine, Nankai University, No. 94 Weijin Road, 300071 Tianjin, China; 2 Department of Occupational and Environmental Health, School of Public Health, Tianjin Medical University, 300070 Tianjin, China; 3 NHC Key Laboratory of Hormones and Development, Tianjin Key Laboratory of Metabolic Diseases, Chu Hsien-I Memorial Hospital & Tianjin Institute of Endocrinology, Tianjin Medical University, 300134 Tianjin, China; 4 Shanghai M-action Health Technology Co., Ltd, 201203 Shanghai, China

**Keywords:** Multimorbidity, Healthy lifestyle, Cognitive impairment, Longitudinal cohort study, Older population

## Abstract

**Background:**

Evidence on the association between multimorbidity and cognitive impairment in Chinese older population is limited. In addition, whether a healthy lifestyle can protect cognitive function in multimorbid older population remains unknown.

**Methods:**

A total of 6116 participants aged ≥ 65 years from the Chinese Longitudinal Healthy Longevity Survey were followed up repeatedly. The number of coexisting chronic diseases was used for assessing multimorbidity and cardiometabolic multimorbidity. Three lifestyle statuses (unhealthy, intermediate, and healthy) were defined based on a lifestyle score covering smoking, alcohol drinking, body mass index, outdoor activities, and dietary pattern. Cognitive impairment was defined as the Mini-Mental State Examination score < 24. A modified Poisson regression model with robust error variance was used to assess the associations between multimorbidity, healthy lifestyle, and cognitive impairment.

**Results:**

During a median follow-up period of 5.8 years, 1621 incident cases of cognitive impairment were identified. The relative risk (RR) of cognitive impairment associated with heavy multimorbidity burden (≥ 3 conditions) was 1.39 (95% confidence interval: 1.22–1.59). This association declined with age, with RRs being 3.08 (1.78–5.31), 1.40 (1.04–1.87), and 1.19 (1.01–1.40) in subjects aged < 70 years, ≥ 70 and < 80 years, and ≥ 80 years, respectively (P for interaction = 0.001). Compared to unhealthy lifestyle, a healthy lifestyle was related to an approximately 40% reduced risk of cognitive impairment regardless of multimorbidity burden. Among the 5 lifestyle factors assessed, daily outdoor activities and a healthy dietary pattern showed convincing protective effects on cognitive function.

**Conclusions:**

The relationship between multimorbidity and cognitive impairment is age-dependent but remains significant in the population aged 80 years or older. A healthy lifestyle may protect cognitive function regardless of the multimorbidity burden. These findings highlight the importance of targeting individuals with heavy multimorbidity burden and promoting a heathy lifestyle to prevent cognitive impairment in Chinese older population.

**Supplementary Information:**

The online version contains supplementary material available at 10.1186/s12889-023-17551-1.

## Introduction

Cognitive impairment is a prevalent condition in older population, characterized by a decline in the ability to think, reason, remember, and communicate effectively [1]. The severity of cognitive impairment can progress from mild cognitive impairment to Alzheimer’s disease and dementia over time [1]. It is estimated that approximately 15.6% of individuals aged 50 years or older have mild cognitive impairment [2], and more than 55 million people worldwide suffer from dementia currently, over 60% of whom live in low- and middle-income countries [3]. To address the significant disease burden caused by cognitive impairment [4], it is crucial to identify high-risk populations and modifiable risk factors associated with cognitive impairment [5].

Multimorbidity, the coexistence of two or more chronic conditions, is a common phenomenon in older adults, particularly in the population aged 80 years or older [6, 7]. Multimorbidity is associated with functional decline, decreased quality of life, and increased healthcare utilization and costs [6]. There is growing evidence that multimorbidity is also associated with cognitive impairment and dementia [8–11]. In addition, cardiometabolic multimorbidity, which refers to the accumulation of cardiovascular and metabolic conditions, is particularly relevant to accelerated cognitive decline and increased risk of dementia [12–14].

However, there are several significant evidence gaps that should be addressed. First, much of the research in this field has been conducted in high-income western countries [8–10, 12–16], and there is a lack of studies in low- and middle-income countries like China [17, 18]. Second, few studies have investigated the association between multimorbidity and cognitive impairment in the population aged 80 years or older, and the findings from these studies are inconclusive [9, 13]. Whether multimorbidity is linked to higher risk of cognitive impairment in the people aged ≥ 80 years remains unclear. Moreover, healthy lifestyle behaviors such as physical activity, a healthy diet, and not smoking have shown protective effects against cognitive impairment and dementia [19]. However, evidence on the impacts of healthy lifestyle factors on cognitive function considering the status of multimorbidity in older population is limited.

China is about to face an unprecedented challenge in managing an increasingly aging population in the next two decades [20]. Addressing the current evidence gaps is crucial for developing effective strategies to prevent and manage cognitive impairment among older adults with multimorbidity. In this study, we utilized data from the Chinese Longitudinal Healthy Longevity Survey (CLHLS) to explore associations between multimorbidity, healthy lifestyle status, and the risk of incident cognitive impairment in Chinese older adults, with a particular emphasis on the population aged 80 years or older.

## Methods

### Study population

The CLHLS is a nationwide community-based cohort study focusing on older adults in China [21]. Up to present, the CLHLS conducted eight waves of surveys in half of the counties and cities randomly selected in 23 provinces across China since 1998 [22]. In each sampled county or city, all centenarians who were willing to participate were included in the study. Then, one octogenarian and one nonagenarian living near each participating centenarian were randomly sampled. In addition, for every three included centenarians, four younger older people (65–79 years old) living nearby were randomly selected since 2002. In each survey, a variety of data including sociodemographic characteristics, lifestyle, cognitive status, psychological and physical conditions, and survival status were collected. A more detailed description of the CLHLS was reported elsewhere [21, 23]. Because adequate data on number of coexisting chronic diseases were collected since 2008, the current study included all participants of 2008 survey (N = 16,954), and newly enrolled subjects in 2011 (N = 1340) and 2014 (N = 1125) surveys. The most recent survey was conducted in 2018. Participants were followed multiple times until they were first identified as having cognitive impairment, dying, or the 2018 survey, whichever came first. Participants with missing Mini-Mental State Examination (MMSE) score, MMSE score < 24, self-reported diagnosis of dementia, or age < 65 at enrollment, and participants who lost or died before the first follow-up or had missing MMSE score during follow-up were excluded. Finally, a total of 6116 subjects with 16,906 observations were included for analyses. The flowchart for inclusion and exclusion of study participants is shown in Supplemental Figure S1.

The CLHLS study was approved by the Ethics Committee of Peking University (IRB00001052-13074). All participants or their representatives signed consent form before data collection.

### Assessment of multimorbidity

Multimorbidity of chronic diseases was assessed by the number of coexisting chronic diseases out of 13 chronic conditions (Table 1). These chronic conditions were selected from the Chinese Multimorbidity Weighted Index and moderately modified to fit the CLHLS dataset [24]. Notably, participants with dementia at baseline were excluded as dementia was considered as a severe stage of cognitive impairment. Therefore, dementia was not included for assessing multimorbidity. In addition, we estimated cardiometabolic multimorbidity using the number of coexisting cardiometabolic diseases including hypertension, dyslipidemia, heart disease, stroke, and diabetes. Ascertainment of the existence of chronic diseases in CLHLS was mainly based on self-reported physician diagnosis of 24 chronic diseases obtained in each survey. In addition, participants with the mean of two systolic/diastolic blood pressure measures ≥ 140/90 mmHg were also treated as hypertension patients even if they reported no physician diagnosis. Besides, in 2008, 2011, and 2014 surveys, anxiety was defined as answering “always” or “often” to the question “Do you fell fearful or anxious?”, while depression was defined as answering “yes” to at least one of the two questions “Have you felt sad, blue, or depressed for two weeks or more in last 12 months?” and “Have you lost interest in most things like hobbies, work, or other activities you usually enjoy for two weeks or more in last 12 months?” [25]. In 2018 survey, anxiety symptoms were assessed using the 7-item Generalized Anxiety Disorder Scale (GAD-7) [26], and depression symptoms were assessed using the 10-item Center for Epidemiological Studies Depression Scale (CESD-10) [27]. Both scales demonstrated satisfactory reliability and validity in Chinese population [26, 27]. We defined anxiety as a score of 5 or above on the GAD-7, and depression as a score of 10 or above on the CESD-10 following earlier studies [26, 28]. Based on data above, the existence of 13 chronic conditions for each participant was determined. A detailed description of the definition and data source of each disease is provided in Table 1.

**Table 1:** Definitions of 13 chronic diseases used for assessment of multimorbidity

Chronic diseases included in the Chinese Multimorbidity Weighted Index [24]	Corresponding chronic diseases defined in the current paper	Data used for definition
Stroke	Stroke or cerebrovascular disease	Self-reported physician diagnosis
Memory-related disease (e.g. dementia, brain atrophy, Parkinson’s disease)	Parkinson’s disease	Self-reported physician diagnosis
Cancer or malignant tumor (excluding minor skin cancers)	Cancer	Self-reported physician diagnosis
Asthma	Asthma or chronic lung disease (bronchitis, emphysema, or pneumonia) *	Self-reported physician diagnosis
Arthritis or rheumatism	Arthritis or rheumatism ^#^	Self-reported physician diagnosis
Emotional, nervous, or psychiatric problems	Anxiety or depression ^#^	Screening questionnaire
Heart disease (e.g. coronary heart disease, angina, congestive heart failure)	Heart disease	Self-reported physician diagnosis
Chronic lung diseases (e.g. chronic bronchitis, emphysema, excluding tumors or cancer)	/	/
Hypertension	Hypertension	Self-reported physician diagnosis; Blood pressure measures
Kidney disease (except for tumor or cancer)	Chronic nephritis	Self-reported physician diagnosis
Diabetes or high blood sugar	Diabetes	Self-reported physician diagnosis
Stomach or other digestive disease	Digestive diseases (peptic ulcers, cholecystitis, or cholelithiasis)	Self-reported physician diagnosis
Dyslipidemia (e.g. elevation of total cholesterol)	Dyslipidemia	Self-reported physician diagnosis
Liver disease (except for fatty liver, tumors, and cancer)	Hepatitis	Self-reported physician diagnosis

*In CLHLS, a single question “Have you been diagnosed with bronchitis, emphysema, asthma, or pneumonia by a physician?” was asked. Therefore, asthma or chronic lung disease (bronchitis, emphysema, or pneumonia) was treated as a single chronic condition.

^#^In 2008 survey, physician diagnosis of rheumatism and the two questions used for screening depression were not asked. Therefore, this information was imputed by data collected in 2011 survey.

### Assessment of healthy lifestyle and other covariates

Data on lifestyle factors and sociodemographic characteristics were obtained from participants or proxy through face-to-face or telephone interview. Missing values of lifestyle factors and other covariates were imputed prior to main analyses. Details in missing values and corresponding dispositions are shown in Supplemental Table S1.

Five lifestyle factors included smoking, alcohol drinking, outdoor activities, dietary pattern, and body mass index. Smoking status was classified as current, former, or never smokers. According to the Chinese Dietary Guidelines 2016, the intake of low-alcohol liquor, high-alcohol liquor, and wine was converted into alcohol intake, respectively [29]. Then drinking status was classified as heavy, moderate, or never drinkers based on total alcohol intake. Heavy drinkers were defined as men who consumed ≥ 25 g of alcohol per day or women who consumed ≥ 15 g of alcohol per day. Moderate drinkers were defined as men who consumed < 25 g of alcohol per day or women who consumed < 15 g of alcohol per day. Who self-reported never having had regular alcohol drinking were considered as never drinkers [30]. The level of outdoor activities was categorized into 3 groups as never, sometimes, or almost daily according to the frequency of engaging in activities like Tai-Chi, plaza dance, socializing with friends, and others. Dietary pattern was assessed by the intake frequency of 5 food categories including fruits, vegetables, fish, bean products, and tea. For intake frequency of “rarely or never”, “sometimes or occasionally”, or “always or almost every day”, a score of 0, 1 or 2 was assigned respectively, with a higher score indicating higher frequency. Then a dietary score was calculated as the sum of the scores of the 5 food categories. Dietary patterns were classified as unfavorable (0 to 3), intermediate (5 to 6), and favorable (7 to 10) based on the dietary score following a previous study [30]. Body mass index (BMI) was calculated as body weight in kilograms divided by height in meters squared. BMI was categorized into 3 groups as underweight (< 18.5 kg/m^2^), normal weight (≥ 18.5 kg/m^2^ and < 24 kg/m^2^), or overweight/obesity (≥ 24 kg/m^2^).

We assigned 0, 1, or 2 points to each group of smoking, alcohol drinking, outdoor activities, and dietary pattern, respectively, with higher points indicating a healthier lifestyle. For BMI, we assigned 2 points to normal weight group and 0 point to both underweight and overweight/obesity group. Subsequently, we summed up the points of 5 lifestyle factors, resulting a healthy lifestyle score ranging from 0 to 10. Based on this score, three healthy lifestyle statuses were defined as healthy (8 to 10), intermediate (6 to 7), and unhealthy (0 to 6) according to a previous study [30].

Covariates included major sociodemographic factors such as age, sex, ethnic group, residential area, marital status, education level, and annul household income. Age was defined as the years between survey dates and verified birth dates. Ethnic group was classified as Han or non-Han Chinese. The classifications of residential area included city, town, and rural area. Marital statuses were classified as married, not married, or widowed. The level of education was divided into no education, less than 9 years, or ≥ 9 years. Annual household income was categorized as low (< 8000 Yuan), medium (≥ 8000 Yuan and < 30,000 Yuan), or high (≥ 30,000 Yuan). In addition, the amount of staple food intake (rice, wheat, coarse cereals, and others) was categorized into three groups according to tertiles and adjusted in sensitivity analysis.

### Assessment of cognitive function

Global cognitive function of participants in CLHSL was assessed by the Chinese version of the MMSE, a widely used assessment tool for measuring cognitive impairment, through face-to-face interview [31]. The Chinese MMSE consists of 24 items that cover 7 domains, including orientation, naming food, registration of 3 words, attention and calculation, copying a figure, recalling, and language capability. The validity and reliability of the Chinese MMSE have been tested and confirmed in previous studies [23, 32]. Participants were given a score between 0 and 30 based on their performance on the test, with higher scores indicating better cognitive function. Based on previous literature [31], we considered participants with an MMSE score below 24 to have cognitive impairment.

### Statistical analysis

Baseline characteristics of study participants were presented as mean ± SD for continuous variables or frequency (percentage) for categorical variables. The difference in baseline characteristics between different age groups was tested using ANOVA or Chi-square tests as appropriate.

The trajectories of the MMSE score along with age were modeled using the generalized additive mixed model [33]. A penalized cubic regression spline of age was added to the model to fit age-MMSE score curves. Subject-specific random intercepts were applied to account for within-subject correlation. In addition, baseline MMSE score, sex, ethnic group, residential area, marital status, education level, and annual household income were included as fixed effects in all models. For models with multimorbidity as the exposure, lifestyle factors including smoking, alcohol drinking, outdoor activities, BMI category, and dietary pattern were also adjusted. For models with healthy lifestyle status as the exposure, the multimorbidity level was adjusted.

We estimated the relative risk (RR) of incident cognitive impairment associated with multimorbidity or healthy lifestyle status using the modified Poisson regression model with robust error variance via generalized estimating equations [34]. Model 1 was adjusted for baseline MMSE score, age, and sex. Model 2 included all covariates mentioned above in addition to model 1. Stratification analyses by age group (≥ 65 years and < 70 years, ≥ 70 years and < 80 years, and ≥ 80 years) or sex were conducted, and a product term was added to the model to test whether interaction effects of age or sex may exist. In addition, the RRs for each chronic condition were calculated to estimate the effect of each disease on the risk of cognitive impairment.

Furthermore, the RRs of incident cognitive impairment in relation to healthy lifestyle status were estimated after stratification by the level of multimorbidity. To further elucidate the potential impacts of age on the associations between multimorbidity, healthy lifestyle status and cognitive impairment, we classified participants into two age groups (≥ 65 years and < 80 years vs. ≥80 years), and performed the stratification analysis within each age group, respectively.

Two sensitivity analyses were conducted to assess the robustness of our results. First, a complete case analysis within participants without missing data was performed. We reanalyzed the association between multimorbidity and cognitive impairment, and the relationship between healthy lifestyle status and cognitive impairment stratified by multimorbidity levels and further stratified by age. Second, we further adjusted the intake of staple food (rice, wheat, coarse cereals, and others) upon model 2.

Statistical analyses were performed using SAS 9.4 (SAS Institute, Cary, NC) or R (version 4.0.1). All tests were two-sided with statistical significance set at P < 0.05.

## Results

### Characteristics of study participants

We observed 1621 incident cases of cognitive impairment out of 6116 participants during a median follow-up period of 5.8 years (interquartile range: 3.4 to 9.9). Table 2 shows the baseline characteristics. The mean age of participants was 79.13 years (SD: 9.58), among whom 53.07% were men and 46.5% were 80 years or older. The data revealed that 26.32%, 38.57%, 22.09%, and 13.02% of participants had 0, 1, 2, or ≥ 3 coexisting chronic diseases at baseline, respectively. Older participants were more likely to be widowed, possessed lower education level, had higher annual household income, and scored lower on the MMSE test. Notably, participants aged ≥ 80 years were less likely to be affected by multimorbidity (≥ 2 conditions) and had lower frequency of outdoor activities than those aged 70 to 80 years. With the increase of age, the prevalence of current smoking and heavily drinking decreased, while the proportion of low body weight (< 18.5 kg/m^2^) and unfavorable dietary pattern increased. The differences in the profiles of multimorbidity and lifestyle between different age groups underscore the necessity to investigate the age-specific relationships between multimorbidity, lifestyle, and cognitive impairment.

**Table 2:** Baseline characteristics of study participants

Variables	Overall (n = 6116)	65 ≤ Age < 70 (n = 1168)	70 ≤ Age < 80 (n = 2104)	Age ≥ 80 (n = 2844)	P value
Age, mean ± SD	79.13 ± 9.58	67.06 ± 1.44	74.18 ± 2.84	87.74 ± 6.21	< 0.001
Men, n (%)	3246(53.07)	633(54.20)	1159(55.09)	1454(51.13)	0.015
Han Chinese, n (%)	5655(92.46)	1083(92.72)	1958(93.06)	2614(91.91)	0.297
**Residential area, n (%)**					
City	1022(16.71)	209(17.89)	348(16.54)	465(16.35)	
Town	1241(20.29)	206(17.64)	433(20.58)	602(21.17)	0.137
Rural	3853(63.00)	753(64.47)	1323(62.88)	1777(62.48)	
**Marital status, n (%)**					
Married	3296(53.99)	935(80.05)	1385(66.05)	976(34.37)	
Not married	67(1.10)	24(2.05)	22(1.05)	21(0.74)	< 0.001
Widowed	2742(44.91)	209(17.89)	690(32.90)	1843(64.89)	
**Education level, n (%)**					
None	2883(47.34)	336(28.84)	905(43.16)	1642(58.06)	
Less than 9 years	2589(42.51)	624(53.56)	964(45.97)	1001(35.40)	< 0.001
≥ 9 years	618(10.15)	205(17.60)	228(10.87)	185(6.54)	
**Annul household income, n (%)**					
Low (< 8000 Yuan)	2275(39.54)	405(36.23)	845(42.33)	1025(38.84)	
Medium (≥ 8000 & <30,000 Yuan)	2356(40.95)	503(44.99)	783(39.23)	1070(40.55)	0.002
High (≥ 30,000 Yuan)	1122(19.50)	210(18.78)	368(18.44)	544(20.61)	
MMSE score, mean ± SD	28.16 ± 1.77	28.85 ± 1.43	28.36 ± 1.65	27.73 ± 1.88	< 0.001
**Number of coexisting chronic diseases, n (%)**					
0	1610(26.32)	335(28.68)	499(23.72)	776(27.29)	< 0.001
1	2359(38.57)	416(35.62)	821(39.02)	1122(39.45)	
2	1351(22.09)	242(20.72)	491(23.34)	618(21.73)	
≥ 3	796(13.02)	175(14.98)	293(13.93)	328(11.53)	
**Smoking, n (%)**					
Current	1441(23.56)	364(31.16)	555(26.38)	522(18.35)	
Former	948(15.50)	143(12.24)	362(17.21)	443(15.58)	< 0.001
Never	3727(60.94)	661(56.59)	1187(56.42)	1879(66.07)	
**Alcohol drinking, n (%)**					
Heavy drinker	757(12.41)	196(16.85)	273(13.01)	288(10.16)	
Moderate drinker	529(8.67)	105(9.03)	191(9.10)	233(8.22)	< 0.001
Non-drinker	4812(78.91)	862(74.12)	1635(77.89)	2315(81.63)	
**Outdoor activities, n (%)**					
Never	1460(23.88)	274(23.46)	429(20.40)	757(26.62)	
Sometimes	1297(21.21)	222(19.01)	436(20.73)	639(22.47)	< 0.001
Almost daily	3358(54.91)	672(57.53)	1238(58.87)	1448(50.91)	
**BMI category, n (%)**					
< 18.5 kg/m^2^	1358(22.37)	169(14.53)	362(17.29)	827(29.40)	
≥ 18.5 kg/m^2^ & <24 kg/m^2^	3497(57.61)	650(55.89)	1251(59.74)	1596(56.74)	< 0.001
≥ 24 kg/m^2^	1215(20.02)	344(29.58)	481(22.97)	390(13.86)	
**Dietary pattern, n (%)**					
Unfavorable	1798(29.45)	300(25.73)	589(28.03)	909(32.02)	
Intermediate	2654(43.47)	510(43.74)	888(42.27)	1256(44.24)	< 0.001
Favorable	1654(27.09)	356(30.53)	624(29.70)	674(23.74)	
**Healthy lifestyle status, n (%)**					
Unhealthy	1800(29.80)	393(34.00)	592(28.39)	815(29.11)	
Intermediate	2285(37.82)	419(36.25)	800(38.37)	1066(38.07)	0.013
Healthy	1956(32.38)	344(29.76)	693(33.24)	919(32.82)	

Missing rates were 0.18% for marital status, 0.43% for education level, 5.94% for household income, 0.29% for alcohol drinking, 0.02% for outdoor activities, 0.75% for BMI category, 0.16% for diet pattern, and 1.23% for healthy lifestyle profile.

*Differences between groups were tested by ANOVA or Chi-square test as appropriate.

BMI, body mass index; MMSE, Mini-Mental State Examination; SD, standard deviation

### MMSE score in relation to multimorbidity and healthy lifestyle

**Fig. 1:**
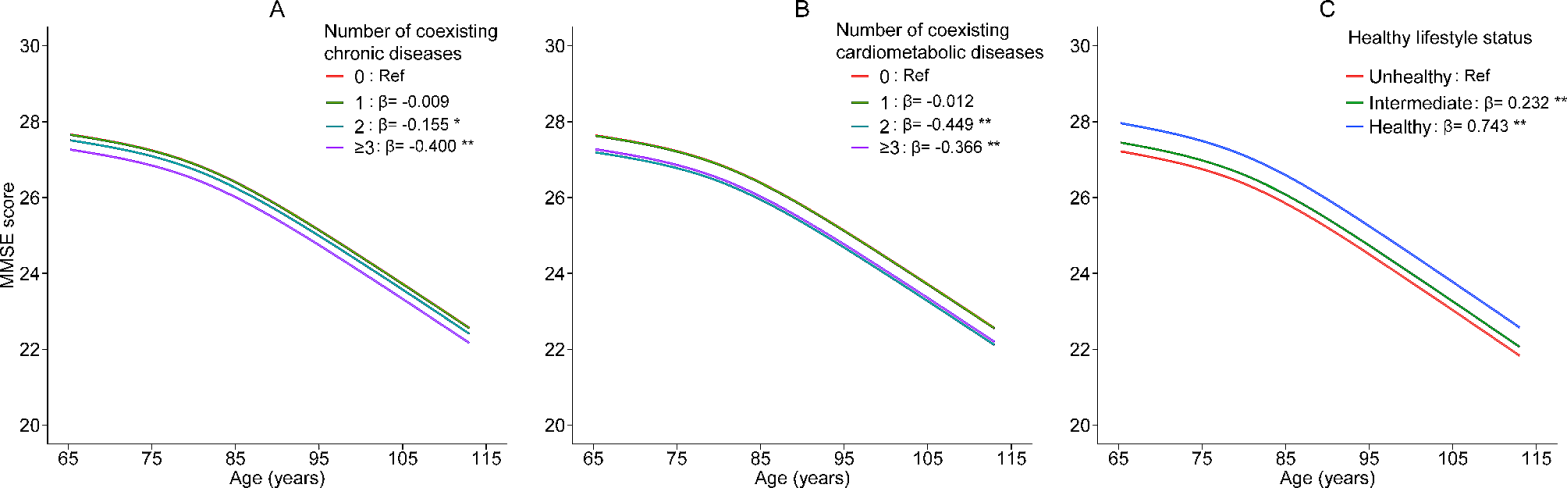
Trajectories of the MMSE score along with age stratified by the level of multimorbidity of chronic diseases (**A**), cardiometabolic multimorbidity (**B**) or healthy lifestyle status (**C**) based on the generalized additive mixed model. Penalized cubic regression spline of age was added in the models to fit the age-MMSE score curves. MMSE, Mini-mental State Examination. * P < 0.05, ** P < 0.001 when compared to reference group

The descending trajectories of the MMSE score against age, stratified by the level of multimorbidity of chronic diseases, cardiometabolic multimorbidity or healthy lifestyle status, were presented in Fig. 1. Compared to no chronic diseases, participants with 2 or ≥ 3 coexisting chronic diseases had significantly lower MMSE score (β= -0.155, P = 0.048 and β= -0.400, P < 0.001, respectively) in a multivariable-adjusted model. Similarly, the MMSE score was also significantly lower in participants with 2 or ≥ 3 coexisting cardiometabolic diseases (β= -0.449, P < 0.001 and β= -0.366, P = 0.002, respectively). Moreover, compared to unhealthy lifestyle style, intermediate and healthy lifestyle were associated with significantly higher MMSE score (β = 0.232, P < 0.001 and β = 0.743, P < 0.001, respectively).

### Multimorbidity and the risk of cognitive impairment, and the role of age

Table 3 shows that there is a gradient increased risk of incident cognitive impairment with the accumulation of both chronic diseases and cardiometabolic diseases. In the multivariable-adjusted analysis, participants with 2, or ≥ 3 coexisting chronic diseases had RRs of cognitive impairment being 1.14 (95% CI: 1.00-1.30, P = 0.058), and 1.39 (95% CI: 1.22–1.59, P < 0.001), compared to no chronic disease. Using the number of coexisting chronic diseases as a continuous variable, a 4% increased risk of cognitive impairment (RR: 1.04, 95% CI: 1.03–1.06, P < 0.001) was observed with each one additional disease. Cardiometabolic multimorbidity was particularly noteworthy in its association with cognitive impairment as evidenced by an 11% increased risk (RR: 1.11, 95% CI: 1.07–1.15, P < 0.001) for each one additional disease. The RRs derived from the multivariable-adjusted model is presented in Supplementary Table S2. We also examined the association between each chronic condition and cognitive impairment. The top 5 chronic conditions that were significantly associated with the cognitive impairment were stroke or cerebrovascular disease, cancer, diabetes, anxiety or depression, and Parkinson’s disease (RRs being 1.54, 1.32, 1.32, 1.30, and 1.27, respectively; all P-values < 0.05) (Supplementary Figure S2).

**Table 3:** Relative risk of incident cognitive impairment associated with multimorbidity

Multimorbidity	Number of cases	Number of observations	Model 1			Model 2	
			RR (95% CI)	P value		RR (95% CI)	P value
**Number of coexisting chronic diseases**							
0	348	3801	Ref			Ref	
1	563	6125	1.01 (0.89–1.14)	0.921		1.02 (0.90–1.14)	0.802
2	345	3646	1.13 (0.99–1.30)	0.073		1.14 (1.00-1.30)	0.058
≥ 3	365	3334	1.35 (1.18–1.54)	< 0.001		1.39 (1.22–1.59)	< 0.001
As a continuous variable	/	/	1.04 (1.02–1.05)	< 0.001		1.04 (1.03–1.06)	< 0.001
**Number of coexisting cardiometabolic diseases**							
0	549	6171	Ref			Ref	
1	682	7218	1.02 (0.92–1.13)	0.713		1.03 (0.93–1.14)	0.604
2	215	2002	1.37 (1.18–1.58)	< 0.001		1.41 (1.22–1.63)	< 0.001
≥ 3	175	1515	1.40 (1.19–1.63)	< 0.001		1.52 (1.30–1.78)	< 0.001
As a continuous variable	/	/	1.09 (1.05–1.13)	< 0.001		1.11 (1.07–1.15)	< 0.001

Model 1: Adjusted for baseline Mini-Mental State Examination score, age, and sex.

Model 2: Further adjusted for ethnic group, residential area, marital status, education level, household income, smoking, alcohol drinking, outdoor activity, body mass index category, and dietary pattern in addition to Model 1.

CI, confidence interval; RR, relative risk

**Fig. 2:**
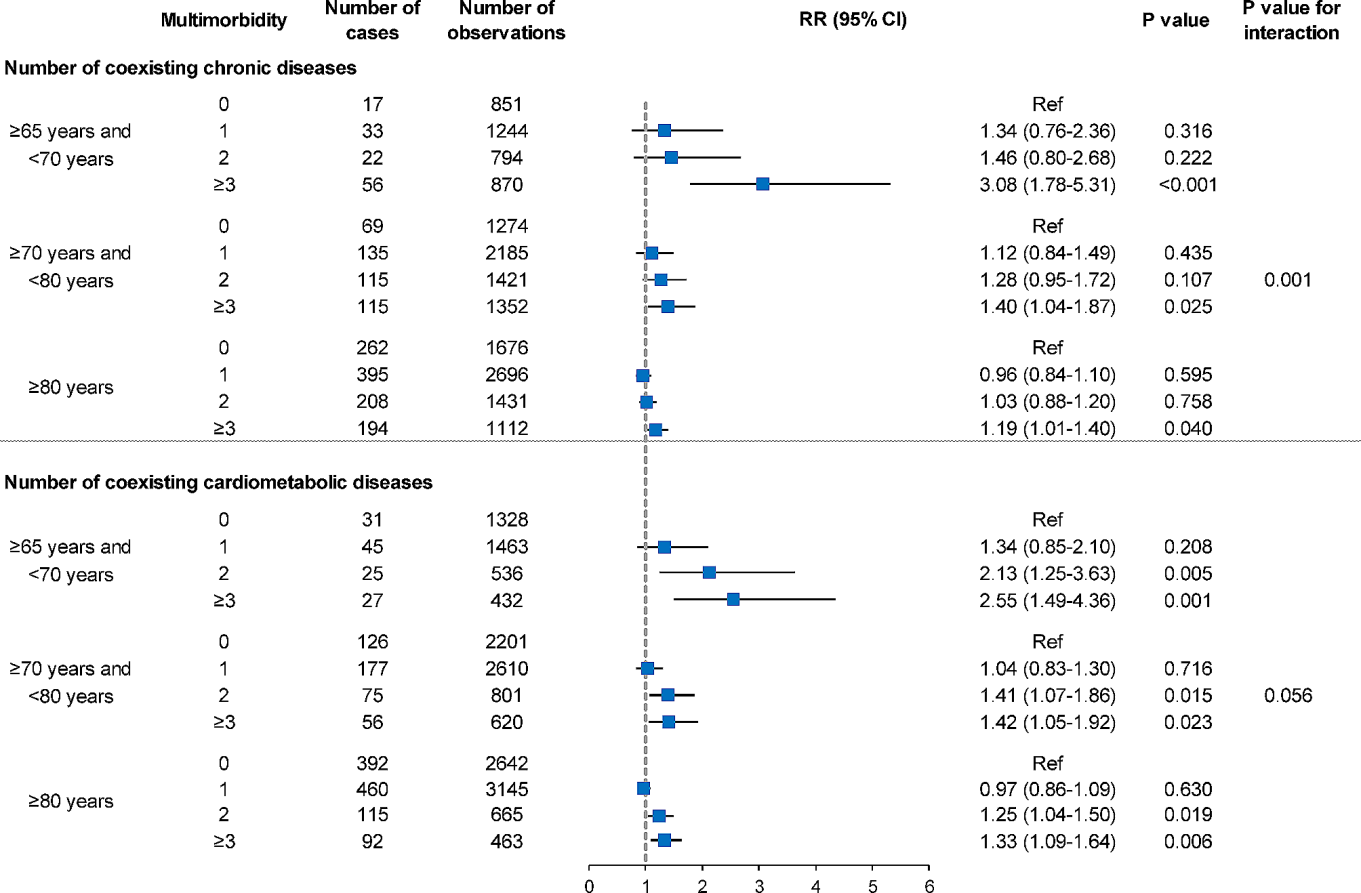
Relative risk of incident cognitive impairment associated with multimorbidity stratified by age. Baseline Mini-Mental State Examination score, age, sex, ethnic group, residential area, marital status, education level, household income, smoking, alcohol drinking, outdoor activities, body mass index category, and dietary pattern were adjusted in the models. CI, confidence interval; RR, relative risk

Stratification analysis based on age groups is presented in Fig. 2. We observed significant interaction effects of age between multimorbidity levels and cognitive impairment (P for interaction = 0.001). The RRs for participants aged 65 to 70 years, 70 to 80 years, and over 80 years with ≥ 3 coexisting chronic diseases were 3.08 (95% CI: 1.78–5.31, P < 0.001), 1.40 (95% CI: 1.04–1.87, P = 0.025), and 1.19 (95% CI: 1.01–1.40, P = 0.040), respectively, with no chronic disease as the reference group. Similarly, we also observed a declining trend in the RRs associated with ≥ 3 coexisting cardiometabolic diseases across older age groups. However, no interaction effect of sex between multimorbidity and cognitive impairment was observed. (Supplementary Figure S3).

### Healthy lifestyle, multimorbidity, and the risk of cognitive impairment

**Fig. 3:**
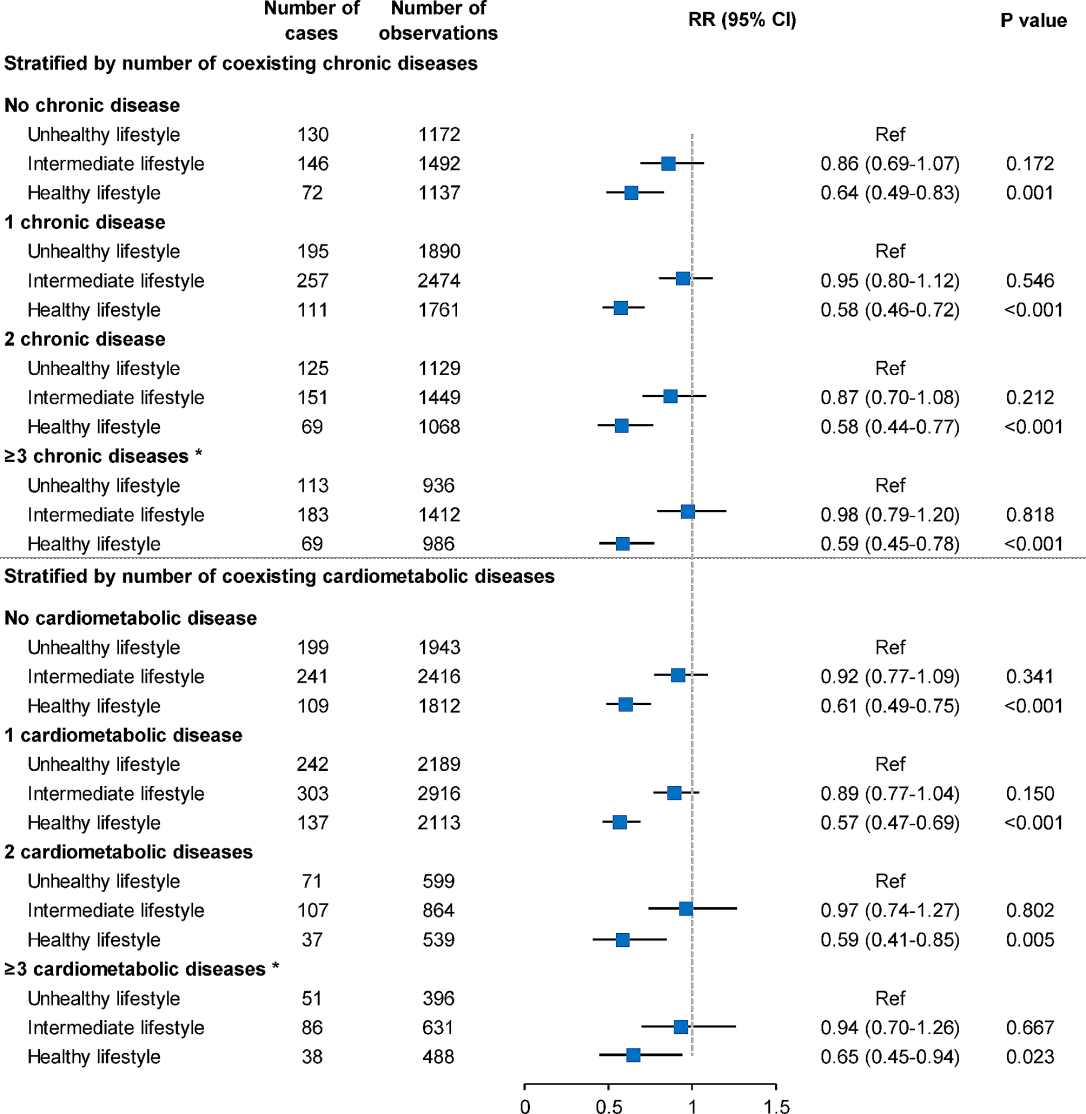
Relative risk of incident cognitive impairment associated with healthy lifestyle status stratified by the level of multimorbidity. Baseline Mini-Mental State Examination score, age, sex, ethnic group, residential area, marital status, education level, and household income were adjusted in the models. *For analyses in this group, the number of coexisting chronic diseases or cardiometabolic diseases was also adjusted. CI, confidence interval; RR, relative risk

**Fig. 4:**
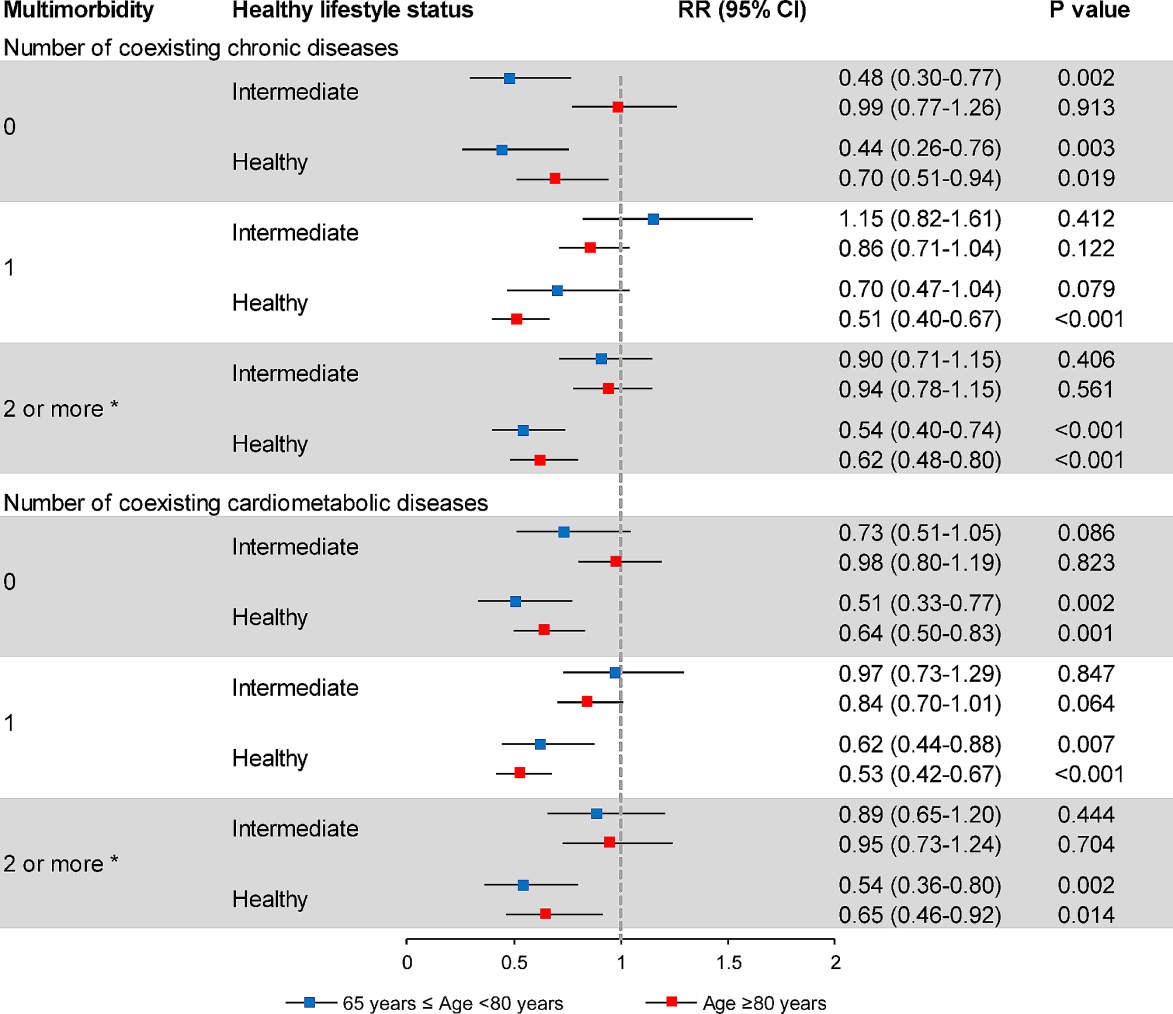
Relative risk of incident cognitive impairment associated with healthy lifestyle status stratified by age and the level of multimorbidity. Unhealthy lifestyle status was used as reference group. Baseline Mini-Mental State Examination score, age, sex, ethnic group, residential area, marital status, education level, and household income were adjusted in the models. *For analyses in this group, the number of coexisting chronic diseases or cardiometabolic diseases was also adjusted. CI, confidence interval; RR, relative risk

Compared to an unhealthy lifestyle, intermediate and healthy lifestyle status were associated with a decreased risk of cognitive impairment, with RRs of 0.92 (95% CI: 0.83–1.01, P = 0.088) and 0.59 (95% CI: 0.52–0.67, P < 0.001), respectively, in multivariable-adjusted analysis of the whole study participants (Supplemental Table S3). In stratification analysis based on multimorbidity level, we noticed an approximately 40% reduced risk of cognitive impairment associated with a healthy lifestyle, regardless of the number of coexisting chronic diseases (Fig. 3). As for individual lifestyle factor, we found that a higher frequency of outdoor activities and a favorable dietary pattern were associated with a reduced risk of cognitive impairment. On the other hand, smoking and alcohol drinking were not found to be statistically associated with cognitive impairment, while overweight/obesity was related to reduced risk of cognitive impairment in the population with heavy multimorbidity burden (≥ 3 conditions) (Supplemental Table S4). Further stratification by multimorbidity level and age group revealed that among individuals with multimorbidity, the protective effect of a healthy lifestyle against cognitive impairment remained significant in the population aged ≥ 80 years (Fig. 4).

### Sensitivity analysis

In the complete case analysis, the positive association between multimorbidity and cognitive impairment remained significant, albeit to a lesser extent (Supplemental Table S5). Moreover, the protective effect of a healthy lifestyle on cognitive impairment in populations with different multimorbidity levels was comparable to that in the main analysis under most circumstances, including those aged ≥ 80 years (Supplemental Figures S4 and S5). In addition, further adjustment for staple food intake had little or no effect on the associations between multimorbidity, healthy lifestyle, and cognitive impairment (Supplemental Table S6).

## Discussion

In this longitudinal cohort study, we observed that multimorbidity, including cardiometabolic multimorbidity, was significantly associated with cognitive decline and an increased risk of cognitive impairment in Chinese older adults. Although the associations were attenuated in older age groups, the risk of cognitive impairment remained significant among the population aged 80 years or older. Importantly, our results suggest that adherence to a healthy lifestyle, particularly frequent daily outdoor activities, and a healthy dietary pattern, was associated with a lower risk of cognitive impairment regardless of the number of chronic diseases. These findings underscore the need for prioritizing prevention programs against cognitive impairment in older adults with heavy multimorbidity burden, while highlighting the potential benefits of preventing major chronic diseases and promoting a healthy lifestyle in reducing the burden of cognitive impairment in all age groups, including the population aged ≥ 80 years.

The positive association between multimorbidity and cognitive function has been reported in previous longitudinal cohort studies, which were mostly conducted in high-income regions like the US, Europe, and South Korea [8, 10, 15, 35–38]. However, only a few cross-sectional studies exploring this relationship have been carried out in low- and middle-income countries [17, 18, 39]. Moreover, the methods for cognitive function assessment varied in these studies, including MMSE, the telephone interview for cognitive status, subjective cognitive complaints, and others [8, 10, 17, 18]. This variation has restricted the extrapolation of prior findings to a distinct population. Our longitudinal cohort study is the first conducted in a low- and middle-income country like China, and it demonstrates a positive correlation between multimorbidity and cognitive impairment, evaluated by the Chinese MMSE score. Our findings provide first-hand evidence to develop prevention strategies against cognitive impairment among older Chinese adults in the context of the increasing multimorbidity burden. Moreover, recent findings from large cohorts such as the Health and Retirement Study, UK Biobank, and Whitehall II cohort study revealed a declining trend of multimorbidity-related dementia risk in older age groups [9, 13, 16, 40]. However, only a few studies have estimated this relationship in the population aged ≥ 80 years, and the results are inconclusive [9, 13]. The Health and Retirement Study reported that the hazard ratio (HR) of incident dementia associated with each additional chronic condition diminished from 1.23 (95% CI: 1.14–1.31) in participants aged ≤ 80 years to 1.02 (95% CI: 0.93–1.12) in participants aged > 80 years [9]. A cohort study in Swedish also found unsignificant association of cardiometabolic multimorbidity with cognitive impairment no dementia (HR: 1.57, 95% CI: 0.80–3.08) and incident dementia (HR: 1.50, 95% CI: 0.75–2.99) in participants aged 78 or above due to relative small sample size (n = 662) [13]. Our study found a similar declining trend of RR for cognitive impairment in older age groups in relation to multimorbidity. More importantly, we confirmed that heavy multimorbidity burden (≥ 3 conditions) did relate to a 19% (RR: 1.19, 95% CI: 1.01–1.40) higher risk of cognitive impairment in the population aged ≥ 80 years, and the risk associated with heavy cardiometabolic burden (≥ 3 cardiometabolic conditions) was even higher (RR: 1.33, 95% CI: 1.09–1.64). The greater increase in the risk of cognitive impairment associated with cardiometabolic multimorbidity is supported by findings from the UK biobank, where the dementia risk was found to be highest in the hypertension, diabetes, and coronary heart disease cluster compared to other disease clusters [16]. Taken together, these data highlight the priority of preventing cardiometabolic diseases to delay or reduce the onset of cognitive impairment and dementia in older adults, including the population aged ≥ 80 years.

Among the 13 chronic conditions we assessed, 8 conditions were related to higher risk of cognitive impairment, including stroke or cerebrovascular diseases, cancer, diabetes, anxiety or depression, Parkinson’s disease, dyslipidemia, heart disease, and hypertension in declining order of RR. The common pathological processes linking cardiovascular diseases, diabetes, and vascular factors such as hypertension and dyslipidemia to cognitive impairment include insults of cerebral vasculature, disruption of blood-brain barrier integrity, neuroinflammation, and Alzheimer pathology such as amyloid deposition [41–44]. Particularly, cerebrovascular lesions caused by stroke can lead to adverse cognitive outcomes through dysregulation of cerebral blood flow, inflammation, and activation of neurodegenerative pathways [42]. Heart diseases including atrial fibrillation, ischemic heart disease, and heart failure could lead to cerebral hypoperfusion and disruption of the blood-brain barrier integrity, which increase susceptibility to neurological insults such as inflammation and amyloid [43]. Chronic hyperglycemia has been linked to changes in white matter microstructure and connectivity, increased occurrence of lacunes, accumulation of advanced glycation end products, and increased blood-brain barrier permeability, which can all contribute to cognitive deficits [44]. Apart from cardiometabolic diseases, depression and anxiety have also been related to cognitive impairment [45–47]. The mechanisms behind this association are complex and could involve hypercortisolism, oxidative stress and inflammation, and reduced functionality of monoamine neurotransmitters induced by chronic psychological distress [47, 48]. Cognitive impairment can occur at any stage of Parkinson’s disease. Although the underlying mechanisms remain largely unclear, cortical involvement of Lewy body and Alzheimer-type pathologies are key features [49]. Finally, the cancer-related cognitive impairment could be attributed to neurological damage caused by cancer treatment, psychological stress of cancer patients, and cancer itself [50].

In addition to multimorbidity, modifiable lifestyle behaviors are well-recognized approaches to prevent cognitive impairment [19]. Our study found an approximately 40% reduced risk of cognitive impairment in relation to a healthy lifestyle, irrespective of multimorbidity burden. Of the 5 lifestyle factors assessed, daily outdoor activities (including both physical and social activities) and a healthy diet showed convincing protective effects, consistent with previous cohort and intervention studies [19, 51]. Besides, overweight/obesity might relate to lower risk of cognitive impairment in population with heavy multimorbidity, which is supported by the fact that lower BMI at late life (> 75 years) is associated with higher risk of cognitive impairment, although the reverse causality cannot be ruled out in this case [19]. Smoking and excessive alcohol drinking are well-known risk factors for cognitive impairment, while light to moderate alcohol consumption could be protective [19]. Some studies suggested that the relationships of smoking and alcohol use with cognitive impairment were age-dependent with less pronounced association in older age group [52, 53]. This may explain our results showing no significant relationship of smoking and alcohol drinking with cognitive impairment, given the relatively large proportion of the population aged ≥ 80 years in our study. More importantly, our study stands out from existing observational studies by focusing on the impact of multimorbidity on the relationship between lifestyle and cognitive impairment. Our findings provide crucial evidence that adhering to a healthy lifestyle, particularly a healthy diet and frequent daily outdoor activities, can protect against cognitive impairment, even in the heavily multimorbid population. Given the increased vulnerability of heavily multimorbid individuals to cognitive impairment, our analysis has significant public health importance and provides a necessary basis for designing lifestyle intervention trials targeting this subgroup. In addition, we found that healthy lifestyle remained protective against cognitive impairment in the population aged ≥ 80 years with a heavy multimorbidity burden, which is particularly relevant given the rapid aging of the population in China in the upcoming decades [20].

This study has several strengths including a longitudinal cohort design with repeated measurements of multimorbidity, lifestyle factors, and cognitive function; a high proportion of the population aged ≥ 80 years (46.5%); and a focus on the impact of multimorbidity on the relationship between healthy lifestyle status and cognitive impairment. However, several limitations must be considered when interpreting our findings. First, we relied on self-reported diagnosis of chronic diseases to assess multimorbidity, which may have introduced recall bias and misclassification. Second, the healthy lifestyle score described in our study was based on previous studies but has not been validated yet. Third, rather than comprehensive clinical evaluations, we used the Chinese MMSE score with a threshold less than 24 to identify cognitive impairment, which could cause bias due to misclassification or false positive diagnosis of cognitive impairment cases. Fourth, selection bias may have been present due to missing MMSE score, loss to follow-up, and death prior to follow-up. Finally, since this study was observational in nature, our findings did not necessarily imply causality. Future studies with more precise assessments of multimorbidity and cognitive impairment are needed to validate our results.

## Conclusions

Our study suggested that multimorbidity was associated with increased risk of incident cognitive impairment in older Chinese including the population aged 80 years or older. We also found that adherence to a healthy lifestyle protected cognitive function regardless of the level of multimorbidity. These findings highlight the importance of targeting individuals with heavy multimorbidity burden and promoting a heathy lifestyle to prevent cognitive impairment in Chinese older population, including the population aged ≥ 80 years.

### Electronic supplementary material

Below is the link to the electronic supplementary material.

Supplementary Material 1

## Data Availability

The datasets supporting the conclusions of this article are available in the Peking University Open Research Data Platform, V2. 10.18170/DVN/WBO7LK.

## Abbreviations

ANOVA: Analysis of variance
BMI: Body mass index
CESD-10: 10-item Center for Epidemiological Studies Depression Scale
GAD-7: 7-item Generalized Anxiety Disorder Scale
CLHLS: Chinese Longitudinal Healthy Longevity Survey
MMSE: Mini-Mental State Examination
