# Supplementary material for: Multimorbidity, healthy lifestyle, and the risk of cognitive impairment in Chinese older adults: a longitudinal cohort study
Source: BMC Public Health. 2024 Jan 2;24:46. doi: 10.1186/s12889-023-17551-1 (PMC10762941; doi:10.1186/s12889-023-17551-1)
Supplement: Supplementary file 1 — Supplementary Material 1 [file 12889_2023_17551_MOESM1_ESM.docx]

**Supplementary Material**

Xing et al. Multimorbidity, Healthy Lifestyle, and the Risk of Cognitive Impairment in Chinese Older Adults: A Longitudinal Cohort Study

**Contents**

**Ⅰ. Supplementary Tables**

**Table S1.** Number of missing values and corresponding dispositions.

**Table S2.** Relative risk of incident cognitive impairment associated with covariates in model 2.

**Table S3.** Relative risk of incident cognitive impairment associated with healthy lifestyle status.

**Table S4.** Relative risk of incident cognitive impairment associated with each lifestyle factor stratified by level of multimorbidity.

**Table S5.** Relative risk of incident cognitive impairment associated with multimorbidity in complete case analysis.

**Table S6.** Relative risk of incident cognitive impairment associated with covariates in model 2 and staple food intake.

**Ⅱ. Supplementary Figures**

**Figure S1.** Inclusion and exclusion of study participants.

**Figure S2.** Relative Risk of incident cognitive impairment associated with each chronic disease.

**Figure S3.** Relative risk of incident cognitive impairment associated with multimorbidity stratified by sex.

**Figure S4.** Relative risk of incident cognitive impairment associated with healthy lifestyle status stratified by the level of multimorbidity in complete case analysis.

**Figure S5.** Relative risk of incident cognitive impairment associated with healthy lifestyle status stratified by age and the level of multimorbidity in complete case analysis.

**Table S1.** Number of missing values and corresponding dispositions.

| **Variables** | **All observations** | **Number of missing observations** | **Percentage of missing observations** | **Disposition** |
| --- | --- | --- | --- | --- |
| Marital status | 16906 | 25 | 0.15% | Mode imputation |
| Education level | 16906 | 56 | 0.33% | Mode imputation |
| Household income | 16906 | 994 | 5.88% | Mode imputation |
| Smoking | 16906 | 39 | 0.23% | Mode imputation |
| Alcohol drinking | 16906 | 148 | 0.88% | Mode imputation |
| Outdoor activities | 16906 | 29 | 0.17% | Mode imputation |
| BMI category | 16906 | 489 | 2.89% | Mode imputation |
| Dietary pattern | 16906 | 171 | 1.01% | Mode imputation |

BMI, body mass index.

**Table S2.** Relative risk of incident cognitive impairment associated with covariates in model 2.

| **Variables** | **RR (95% CI)** | **P value** |
| --- | --- | --- |
| **Number of coexisting chronic diseases** |  |  |
| 0 | Ref |  |
| 1 | 1.02 (0.90-1.14) | 0.802 |
| 2 | 1.14 (1.00-1.30) | 0.058 |
| ≥3 | 1.39 (1.22-1.59) | <0.001 |
| **Baseline MMSE score** | 0.95 (0.92-0.97) | <0.001 |
| **Age, years** | 1.06 (1.06-1.07) | <0.001 |
| **Sex** (1=Men, 0=Women) | 0.77 (0.69-0.87) | <0.001 |
| **Han Chinese** (1=Yes, 0=No) | 1.09 (0.93-1.28) | 0.293 |
| **Marital status** |  |  |
| Married | Ref |  |
| Not married | 0.97 (0.59-1.59) | 0.903 |
| Widowed | 1.03 (0.92-1.14) | 0.635 |
| **Residential area** |  |  |
| City | Ref |  |
| Town | 0.97 (0.83-1.14) | 0.729 |
| Rural | 1.04 (0.91-1.19) | 0.581 |
| **Education level** |  |  |
| None | Ref |  |
| Less than 9 years | 0.69 (0.62-0.77) | <0.001 |
| ≥9 years | 0.43 (0.33-0.57) | <0.001 |
| **Household income** |  |  |
| Low (<8000 Yuan) | Ref |  |
| Medium (≥8000 & <30,000 Yuan) | 0.99 (0.90-1.08) | 0.760 |
| High (≥30,000 Yuan) | 0.88 (0.77-1.00) | 0.051 |
| **Smoking status** |  |  |
| Current | Ref |  |
| Former | 0.92 (0.77-1.09) | 0.325 |
| Never | 0.97 (0.84-1.11) | 0.660 |
| **Alcohol drinking** |  |  |
| Heavy drinker | Ref |  |
| Moderate drinker | 1.21 (0.94-1.55) | 0.131 |
| Non-drinker | 1.14 (0.95-1.37) | 0.146 |
| **Outdoor activity** |  |  |
| Never | Ref |  |
| Sometimes | 0.76 (0.68-0.86) | <0.001 |
| Almost everyday | 0.57 (0.51-0.63) | <0.001 |
| **BMI category** |  |  |
| <18.5 kg/m^2^ | 1.05 (0.94-1.16) | 0.400 |
| ≥18.5 kg/m^2^ & <24 kg/m^2^ | Ref |  |
| ≥24 kg/m2 | 0.91 (0.80-1.04) | 0.162 |
| **Dietary pattern** |  |  |
| Unfavorable | Ref |  |
| Intermediate | 0.75 (0.69-0.83) | <0.001 |
| Favorable | 0.50 (0.42-0.58) | <0.001 |

BMI, body mass index; CI, confidence interval; MMSE, mini-mental state examination; RR, relative risk.

**Table S3.** Relative risk of incident cognitive impairment associated with healthy lifestyle status.

|  | **Unhealthy** | **Intermediate (6≤lifestyle score≤7)** | **Healthy (8≤lifestyle score≤10)** | **As a continuous variable** |
| --- | --- | --- | --- | --- |
|  | **(0≤lifestyle score≤5)** |  |  |  |
| No of cases | 563 | 737 | 321 | / |
| No of observations | 5127 | 6827 | 4952 | / |
| **Model 1** |  |  |  |  |
| RR (95% CI) | Ref | 0.91 (0.82-1.01) | 0.56 (0.49-0.64) | 0.88 (0.86-0.90) |
| P value |  | 0.066 | <0.001 | <0.001 |
| **Model 2** |  |  |  |  |
| RR (95% CI) | Ref | 0.92 (0.83-1.01) | 0.59 (0.52-0.67) | 0.89 (0.87-0.91) |
| P value |  | 0.088 | <0.001 | <0.001 |

Model 1: Adjusted for baseline Mini-Mental State Examination score, age, and sex.

Model 2: Further adjusted for ethnic group, residential area, marital status, education level, household income, and number of coexisting chronic diseases in addition to Model 1.

CI, confidence interval; RR, relative risk.

**Table S4.** Relative risk of incident cognitive impairment associated with each lifestyle factor stratified by level of multimorbidity.

|  | **Number of coexisting chronic diseases** | | | |  | **Number of coexisting cardiometabolic diseases** | | | |
| --- | --- | --- | --- | --- | --- | --- | --- | --- | --- |
|  | **0** | **1** | **2** | ≥**3*** |  | **0** | **1** | **2** | ≥**3*** |
| **Smoking** |  |  |  |  |  |  |  |  |  |
| Current | Ref | Ref | Ref | Ref |  | Ref | Ref | Ref | Ref |
| Former | 0.92 (0.64-1.34) | 0.82 (0.60-1.11) | 1.10 (0.76-1.60) | 0.90 (0.63-1.30) |  | 0.78 (0.58-1.05) | 0.98 (0.74-1.29) | 1.35 (0.83-2.20) | 0.80 (0.48-1.32) |
| Never | 0.89 (0.67-1.18) | 0.94 (0.74-1.18) | 1.04 (0.75-1.45) | 1.05 (0.77-1.44) |  | 0.85 (0.68-1.07) | 1.02 (0.82-1.28) | 1.34 (0.86-2.07) | 0.92 (0.60-1.41) |
| **Alcohol drinking** |  |  |  |  |  |  |  |  |  |
| Heavy drinker | Ref | Ref | Ref | Ref |  | Ref | Ref | Ref | Ref |
| Moderate drinker | 1.21 (0.70-2.07) | 1.28 (0.86-1.90) | 1.20 (0.66-2.17) | 1.12 (0.64-1.95) |  | 1.21 (0.79-1.85) | 1.54 (1.06-2.23) | 0.45 (0.15-1.35) | 1.13 (0.48-2.65) |
| Non-drinker | 1.28 (0.86-1.92) | 1.15 (0.86-1.53) | 1.27 (0.82-1.96) | 0.95 (0.65-1.39) |  | 1.28 (0.93-1.76) | 1.19 (0.89-1.57) | 0.81 (0.50-1.32) | 1.14 (0.67-1.93) |
| **Outdoor activity** |  |  |  |  |  |  |  |  |  |
| Never | Ref | Ref | Ref | Ref |  | Ref | Ref | Ref | Ref |
| Sometimes | 0.81 (0.63-1.05) | 0.76 (0.62-0.92) | 0.90 (0.70-1.15) | 0.64 (0.50-0.81) |  | 0.86 (0.70-1.05) | 0.77 (0.65-0.92) | 0.74 (0.54-1.01) | 0.56 (0.38-0.81) |
| Almost every day | 0.65 (0.53-0.81) | 0.58 (0.49-0.69) | 0.62 (0.49-0.79) | 0.44 (0.35-0.55) |  | 0.62 (0.52-0.74) | 0.58 (0.50-0.69) | 0.48 (0.35-0.64) | 0.47 (0.35-0.64) |
| **BMI category** |  |  |  |  |  |  |  |  |  |
| <18.5 kg/m^2^ | 1.09 (0.88-1.35) | 1.07 (0.90-1.28) | 1.17 (0.93-1.46) | 0.84 (0.65-1.08) |  | 1.03 (0.87-1.22) | 1.13 (0.96-1.32) | 0.89 (0.63-1.25) | 1.05 (0.73-1.51) |
| ≥18.5 & <24 kg/m^2^ | Ref | Ref | Ref | Ref |  | Ref | Ref | Ref | Ref |
| ≥24 kg/m^2^ | 1.20 (0.89-1.62) | 0.96 (0.77-1.19) | 0.92 (0.70-1.20) | 0.74 (0.58-0.95) |  | 1.20 (0.95-1.52) | 0.83 (0.68-1.01) | 0.84 (0.63-1.14) | 0.73 (0.52-1.03) |
| **Dietary pattern** |  |  |  |  |  |  |  |  |  |
| Unfavorable | Ref | Ref | Ref | Ref |  | Ref | Ref | Ref | Ref |
| Intermediate | 0.83 (0.68-1.02) | 0.72 (0.61-0.85) | 0.67 (0.54-0.83) | 0.80 (0.66-0.98) |  | 0.75 (0.64-0.88) | 0.69 (0.60-0.81) | 0.92 (0.71-1.20) | 0.79 (0.59-1.06) |
| Favorable | 0.53 (0.37-0.77) | 0.42 (0.32-0.56) | 0.45 (0.32-0.64) | 0.60 (0.44-0.83) |  | 0.47 (0.35-0.63) | 0.41 (0.31-0.53) | 0.72 (0.49-1.07) | 0.74 (0.47-1.14) |

Baseline Mini-Mental State Examination score, age, sex, ethnic group, residential area, marital status, education level, household income, smoking, alcohol drinking, outdoor activity, BMI category, and dietary pattern were included in the models.

*For analyses in this group, the number of coexisting chronic diseases or cardiometabolic diseases was also included in the models.

BMI, body mass index; CI, confidence interval; RR, relative risk.

**Table S5.** Relative risk of incident cognitive impairment associated with multimorbidity in complete case analysis.

| **Multimorbidity** | **Number of cases** | **Number of observations** | **Model 1** | |  | **Model 2** | |
| --- | --- | --- | --- | --- | --- | --- | --- |
|  |  |  | **RR (95% CI)** | **P value** |  | **RR (95% CI)** | **P value** |
| **Number of coexisting chronic diseases** | | | | | | | |
| 0 | 294 | 3406 | Ref |  |  | Ref |  |
| 1 | 470 | 5503 | 0.99 (0.87-1.13) | 0.860 |  | 0.99 (0.87-1.13) | 0.848 |
| 2 | 288 | 3253 | 1.12 (0.97-1.31) | 0.124 |  | 1.13 (0.97-1.31) | 0.105 |
| ≥3 | 280 | 2928 | 1.26 (1.08-1.46) | 0.003 |  | 1.32 (1.14-1.53) | <0.001 |
| As a continuous variable |  |  | 1.03 (1.00-1.05) | 0.017 |  | 1.03 (1.01-1.06) | 0.001 |
| **Number of coexisting cardiometabolic diseases** | | | | | | | |
| 0 | 468 | 5523 | Ref |  |  | Ref |  |
| 1 | 558 | 6455 | 0.99 (0.88-1.10) | 0.802 |  | 0.99 (0.89-1.11) | 0.872 |
| 2 | 178 | 1791 | 1.33 (1.13-1.56) | <0.001 |  | 1.40 (1.19-1.64) | <0.001 |
| ≥3 | 128 | 1321 | 1.24 (1.03-1.48) | 0.022 |  | 1.37 (1.14-1.64) | 0.001 |
| As a continuous variable |  |  | 1.06 (1.02-1.11) | 0.003 |  | 1.09 (1.04-1.13) | <0.001 |

Model 1: Adjusted for baseline Mini-Mental State Examination score, age, and sex.

Model 2: Further adjusted for ethnic group, residential area, marital status, education level, household income, smoking, alcohol drinking, outdoor activity, body mass index category, and dietary pattern in addition to Model 1.

CI, confidence interval; RR, relative risk.

**Table S6.** Relative risk of incident cognitive impairment associated with covariates in model 2 and staple food intake.

| **Variables** | **RR (95% CI)** | **P value** |
| --- | --- | --- |
| **Number of coexisting chronic diseases** |  |  |
| 0 | Ref |  |
| 1 | 1.01 (0.90-1.14) | 0.841 |
| 2 | 1.13 (0.99-1.29) | 0.078 |
| ≥3 | 1.36 (1.19-1.56) | <0.001 |
| **Baseline MMSE score** | 0.95 (0.92-0.97) | <0.001 |
| **Age, years** | 1.06 (1.05-1.06) | <0.001 |
| **Sex** (1=Men, 0=Women) | 0.81 (0.72-0.92) | <0.001 |
| **Han Chinese** (1=Yes, 0=No) | 1.11 (0.95-1.31) | 0.182 |
| **Marital status** |  |  |
| Married | Ref |  |
| Not married | 0.96 (0.59-1.56) | 0.859 |
| Widowed | 1.04 (0.94-1.16) | 0.425 |
| **Residential area** |  |  |
| City | Ref |  |
| Town | 0.98 (0.84-1.15) | 0.837 |
| Rural | 1.05 (0.92-1.20) | 0.464 |
| **Education level** |  |  |
| None | Ref |  |
| Less than 9 years | 0.68 (0.61-0.76) | <0.001 |
| ≥9 years | 0.43 (0.32-0.56) | <0.001 |
| **Household income** |  |  |
| Low (<8000 Yuan) | Ref |  |
| Medium (≥8000 & <30,000 Yuan) | 0.98 (0.89-1.07) | 0.626 |
| High (≥30,000 Yuan) | 0.87 (0.76-0.99) | 0.033 |
| **Smoking status** |  |  |
| Current | Ref |  |
| Former | 0.92 (0.78-1.09) | 0.339 |
| Never | 0.96 (0.84-1.10) | 0.552 |
| **Alcohol drinking** |  |  |
| Heavy drinker | Ref |  |
| Moderate drinker | 1.18 (0.92-1.51) | 0.195 |
| Non-drinker | 1.12 (0.94-1.35) | 0.204 |
| **Outdoor activity** |  |  |
| Never | Ref |  |
| Sometimes | 0.77 (0.68-0.86) | <0.001 |
| Almost everyday | 0.58 (0.52-0.64) | <0.001 |
| **BMI category** |  |  |
| <18.5 kg/m^2^ | 1.03 (0.93-1.15) | 0.520 |
| ≥18.5 kg/m^2^ & <24 kg/m^2^ | Ref |  |
| ≥24 kg/m2 | 0.93 (0.82-1.06) | 0.272 |
| **Dietary pattern** |  |  |
| Unfavorable | Ref |  |
| Intermediate | 0.76 (0.69-0.84) | <0.001 |
| Favorable | 0.50 (0.43-0.59) | <0.001 |
| **Tertiles of staple food intake** (rice, wheat, coarse cereals, and others) |  |  |
| First tertile | Ref |  |
| Second tertile | 0.79 (0.72-0.88) | <0.001 |
| Third tertile | 0.63 (0.54-0.73) | <0.001 |

BMI, body mass index; CI, confidence interval; MMSE, mini-mental state examination; RR, relative risk.

**Figure S1.** Inclusion and exclusion of study participants.

**Figure S2.** Relative Risk of incident cognitive impairment associated with each chronic disease.

Baseline Mini-Mental State Examination score, age, sex, ethnic group, residential area, marital status, education level, household income, smoking, alcohol drinking, outdoor activity, body mass index category, and dietary pattern were adjusted in the models.

CI, confidence interval; RR, relative risk.

**Figure S3.** Relative risk of incident cognitive impairment associated with multimorbidity stratified by sex.

Baseline Mini-Mental State Examination score, age, ethnic group, residential area, marital status, education level, household income, smoking, alcohol drinking, outdoor activity, body mass index category, and dietary pattern were adjusted in the models.

CI, confidence interval; RR, relative risk.

**Figure S4.** Relative risk of incident cognitive impairment associated with healthy lifestyle status stratified by the level of multimorbidity in complete case analysis.

Baseline Mini-Mental State Examination score, age, sex, ethnic group, residential area, marital status, education level, and household income were adjusted in the models.

*For analyses in this group, the number of coexisting chronic diseases or cardiometabolic diseases was also adjusted.

CI, confidence interval; RR, relative risk.

**Figure S5.** Relative risk of incident cognitive impairment associated with healthy lifestyle status stratified by age and the level of multimorbidity in complete case analysis.

Unhealthy lifestyle status was used as reference group.

Baseline Mini-Mental State Examination score, age, sex, ethnic group, residential area, marital status, education level, and household income were adjusted in the models. *For analyses in this group, the number of coexisting chronic diseases or cardiometabolic diseases was also adjusted.

CI, confidence interval; RR, relative risk.
